# Assessing the mechanism of response in the retrosplenial cortex of good and poor navigators^☆^

**DOI:** 10.1016/j.cortex.2013.08.002

**Published:** 2013-11

**Authors:** Stephen D. Auger, Eleanor A. Maguire

**Affiliations:** Wellcome Trust Centre for Neuroimaging, Institute of Neurology, University College London, London, UK

**Keywords:** Retrosplenial cortex, Permanence, Landmarks, Navigation, Episodic memory

## Abstract

The retrosplenial cortex (RSC) is consistently engaged by a range of tasks that examine episodic memory, imagining the future, spatial navigation, and scene processing. Despite this, an account of its exact contribution to these cognitive functions remains elusive. Here, using functional MRI (fMRI) and multi-voxel pattern analysis (MVPA) we found that the RSC coded for the specific number of permanent outdoor items that were in view, that is, items which are fixed and never change their location. Moreover, this effect was selective, and was not apparent for other item features such as size and visual salience. This detailed detection of the number of permanent items in view was echoed in the parahippocampal cortex (PHC), although the two brain structures diverged when participants were divided into good and poor navigators. There was no difference in the responsivity of the PHC between the two groups, while significantly better decoding of the number of permanent items in view was possible from patterns of activity in the RSC of good compared to poor navigators. Within good navigators, the RSC also facilitated significantly better prediction of item permanence than the PHC. Overall, these findings suggest that the RSC in particular is concerned with coding the presence of every permanent item that is in view. This mechanism may represent a key building block for spatial and scene representations that are central to episodic memories and imagining the future, and could also be a prerequisite for successful navigation.

## 1 Introduction

The retrosplenial cortex (RSC) comprises Brodmann areas 29/30 and is part of an extended network of brain regions engaged during fMRI studies of autobiographical memory, spatial navigation, imagining fictitious and future experiences and scene processing (Addis, Wong, & Schacter, 2007; Epstein, 2008, 2011; Maguire, 2001a, 2001b; Hassabis, Kumaran, & Maguire, 2007; Spreng, Mar, & Kim, 2009; Svoboda, McKinnon, & Levine, 2006; Troiani, Stigliani, Smith, & Epstein, 2012). RSC is particularly interesting because damage that involves this region in humans can result in significant memory and navigation deficits (Aggleton, 2010; Maguire, 2001b; Vann, Aggleton, & Maguire, 2009), while the earliest metabolic decline in Alzheimer's disease is centred on RSC (Minoshima et al., 1997; Nestor, Fryer, Ikeda, & Hodges, 2003; Pengas, Hodges, Watson, & Nestor, 2010; Villain et al., 2008). Yet despite this, its precise function remains elusive.

In a recent fMRI study by Auger, Mullally, and Maguire (2012) we offered another insight into the role of RSC. We examined different features of items that are normally found outdoors in the everyday environment, including their size, visual salience and the permanence or stability of their location. Participants viewed images of these items one at a time, with RSC responding to only the most permanent, never moving, items. Therefore, even when complex memories, navigation or scenes were not involved, a robust RSC response was evident at the level of single, permanent landmarks. We then examined participants who were good or poor navigators, and found that the latter were much less reliable at identifying the most permanent items. Moreover, when responses to the most permanent items were examined using fMRI, poor navigators had significantly reduced responses in RSC. This suggested that the RSC's contribution may be to provide input regarding permanent items upon which other brain areas can then build effective spatial and scene representations (Auger et al., 2012).

Our previous study (Auger et al., 2012) focussed on single items; however, in the real world, we do not normally encounter items in isolation. In order to promote a proper understanding of the role of the RSC, we need to test its reaction to multiple items, as this will inform whether its responsivity is item-specific or more general. Therefore, the question we addressed here was whether RSC is simply engaged by the presence of permanence per se, irrespective of the number of permanent items being viewed, or whether is it mechanistically more nuanced, tracking the specific number of permanent items. Adjudicating between these two options is important, as going forward it could guide how we conceptualise the function of the RSC and probe the mechanisms that may operate therein. If RSC codes for just the presence of permanence, then its input into spatial and scene representations would be limited. However, if RSC represents each permanent item in a given view, then it could play a key role in detecting and mapping individual landmarks as we encounter them in our surroundings. This operation could be crucial for successful navigation, as the very building blocks of any representation of an environment are the most stable items within it.

**Fig. 1:**
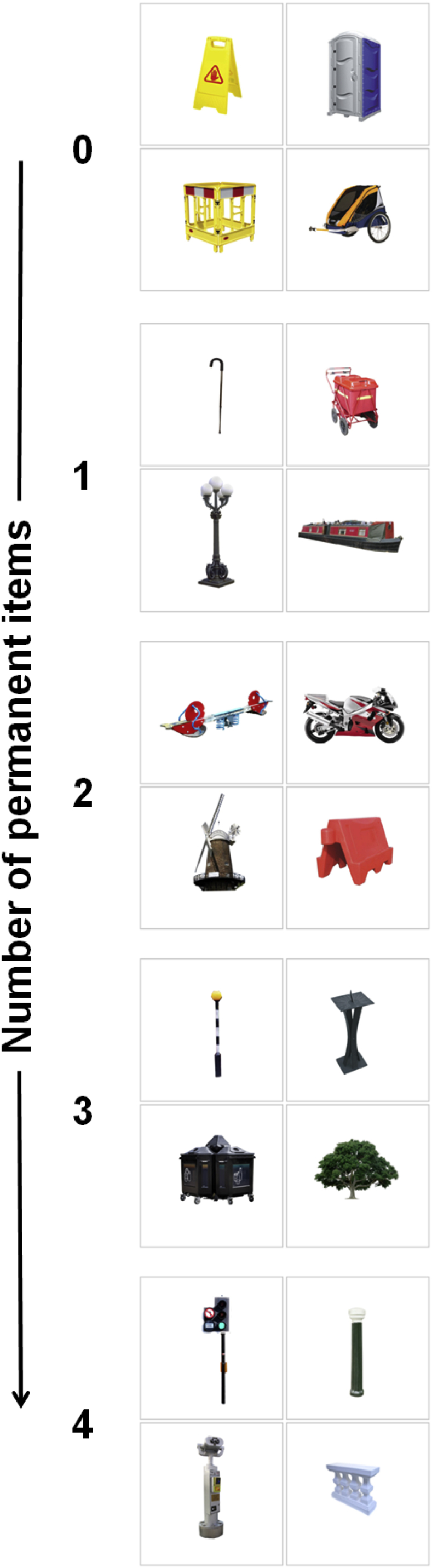
Examples of the stimuli. Categories varied according to the number of permanent, ‘never moving’, items they contained. One example stimulus from each of the five permanence categories is shown here, ranging from no permanent items in the top stimulus, to all four items being permanent in the bottom stimulus.

To test the nature of RSC processing, we had good and poor navigators view quartets of outdoor items (Fig. 1). The stimuli differed in terms of how many of their four items were permanent, i.e., with a fixed location in the environment – they contained either no, 1, 2, 3, or 4 permanent items. We used multi-voxel pattern analysis (MVPA; Chadwick, Bonnici, & Maguire, 2012; Haynes & Rees, 2006; Norman, Polyn, Detre, & Haxby, 2006) to assess whether information about the number of permanent items in view could be decoded from activity in RSC and, if so, whether this differed between good and poor navigators. The quartets were carefully designed such that variations in landmark size and visual salience could be assessed by the same method, allowing us to determine whether any patterns of response observed in RSC were specific to item permanence.

## 2 Materials and methods

### 2.1 Participants

Thirty-two, right-handed, healthy participants (16 females, mean age 23.5 years, SD 2.5) took part in the experiment. All had normal or corrected to normal vision, were highly proficient in English and gave written informed consent in accordance with the local research ethics committee. None of the participants had taken part in any of our previous studies of item permanence.

### 2.2 Stimuli and procedure

Each stimulus comprised four different everyday outdoor items, with each item enclosed by a grey outline on a white background, and laid out in a grid (Fig. 1). The stimuli differed in terms of how many of their four items were permanent – they contained either no, 1, 2, 3, or 4 permanent items (giving 5 category types). Permanent items were defined as those consistently rated as ‘never moving’ by an independent set of participants from previous behavioural experiments (Auger et al., 2012). There were 20 stimuli for each of the 5 category types, giving 100 stimuli in total. We ensured that across the trials of each condition, the non-permanent elements were sampled from the full range of permanence ratings (excluding those that ‘never moved’). The stimuli not only varied according to the number of permanent items they contained; their items also varied in terms of real-world size and visual salience. The size and visual salience of items was also determined by an independent set of participants from the previous behavioural experiments (Auger et al., 2012). In designing the stimuli we ensured a full range of values of these two other landmark features, from the very smallest to largest, and from least to most salient items. This allowed us to also group the 100 stimuli into 5 categories for size and 5 for visual salience. In addition, the stimuli were designed to ensure that a range of size and visual salience values were represented within each permanence category. Overall, therefore, the experimental design allowed us to test the specific effects of item permanence independent of these two other item features. The location of the permanent items within the grid was pseudorandomised to ensure they appeared equally in the 4 possible screen locations. In addition to the 100 stimuli depicting 4 items, there were a further 20 baseline stimuli. These consisted of 4 grey outlines which each contained a black centrally located fixation cross rather than an outdoor item.

Participants were naïve to our interest in item features and believed they were being tested for vigilance and attention. Before entering the scanner, participants were instructed to look closely at all 4 items (or fixation crosses) in each image and to respond with a button press whenever a small blue dot appeared on one of the items (or when a fixation cross turned blue). It was stressed that they should look at all 4 items equally so as to maximise their chances of detecting the blue dots. They were also instructed to focus on the items individually, and not think about any other objects, contexts or personal memories, nor should they link the 4 items together into a scene. Participants then practised the task with stimuli not included in the scanning set.

A typical trial in the scanner consisted of a stimulus being displayed for 6 sec separated by a randomly jittered interval of between 2 and 5 sec during which participants looked at a centrally located black fixation cross on a white background. There were 19 catch trials in addition to the 120 normal trials. During catch trials a small blue dot appeared somewhere on one of the 4 items for 3 sec. Participants were instructed to respond with a button press if they saw a blue dot (or if a fixation cross turned blue in the baseline trials). The order of trials was pseudorandomised ensuring that all stimulus types were distributed across the scanning sessions, of which there were three. No stimuli were repeated.

Immediately after scanning, participants rated how difficult they found the task, and how difficult it was to keep the 4 items separate. Participants also completed several neuropsychological tests: the Rey–Osterrieth Complex Figure (Osterrieth, 1944; Rey, 1941), and the Matrix Reasoning sub-test of the Wechsler Abbreviated Scale of Intelligence (Wechsler, 1999). At the very end of the experiment, participants filled out the Santa Barbara Sense of Direction Scale (SBSOD; Hegarty, Richardson, Montello, Lovelace, & Subbiah, 2002), a self-report questionnaire shown to strongly correlate with navigational ability, and which is increasingly used as a gauge of real-world navigation performance (Auger et al., 2012; Epstein, Higgins, & Thompson-Schill, 2005; Hegarty et al., 2002; Janzen, Jansen, & van Turennout, 2008; Wegman & Janzen, 2011).

### 2.3 Eye-tracking

To assess whether participants attended to all 4 items in the stimuli equally, we recorded their eye movements during fMRI scanning with an MRI-compatible ASL-500 series eye-tracking system (http://www.asleyetracking.com) sampling at 50 Hz.

### 2.4 Scanning details

MRI data were acquired on a 3T Magnetom Allegra head-only MRI scanner (Siemens Healthcare, Erlangen, Germany) operated with the standard transmit-receive head coil. Functional MRI data were acquired in three sessions with a blood oxygenation level-dependent (BOLD) sensitive T2*-weighted single-shot echo-planar imaging sequence which was optimized to minimize signal dropout in the medial temporal lobe (Weiskopf, Hutton, Josephs, & Deichmann, 2006). The sequence used a descending slice acquisition order with a slice thickness of 2 mm, an interslice gap of 1 mm, and an in-plane resolution of 3 × 3 mm. Forty eight slices were collected covering the entire brain, resulting in a repetition time of 2.88 sec. The echo time was 30 msec and the flip angle 90°. All data were acquired at a −45° angle to the anterior–posterior axis. In addition, field maps were collected for subsequent distortion correction (Weiskopf et al., 2006). These were acquired with a double-echo gradient echo field map sequence (TE = 10 and 12.46 msec, TR = 1020 msec, matrix size 64 × 64, with 64 slices, voxel size = 3 mm^3^) covering the whole head. After these functional scans, a 3D MDEFT T1-weighted structural scan was acquired for each participant with 1 mm isotropic resolution (Deichmann, Schwarzbauer, & Turner, 2004). FMRI data were pre-processed using SPM8 (www.fil.ion.ucl.ac.uk/spm). The first 6 ‘dummy’ volumes from each of the three sessions were discarded to allow for T1 equilibration effects. Images were realigned and unwarped (using the field maps) and normalised to a standard EPI template in MNI space with a resampled voxel size of 3 × 3 × 3 mm. Functional data were left unsmoothed for the decoding analyses to facilitate the detection of information present across patterns of voxels. Each trial was modelled as a separate regressor for the 6sec stimulus duration and convolved with the canonical haemodynamic response function. Catch trials were combined into a single regressor and, along with participant-specific movement regressors, were included as covariates of no interest. Participant-specific parameter estimates pertaining to each regressor (betas) were calculated for each voxel.

### 2.5 Regions of interest

Motivated by the findings of Auger et al. (2012), our main region of interest (ROI) was the RSC. In this previous study of item features, we found that the parahippocampal cortex (PHC) responded to permanence as well as to a range of other features (Auger et al., 2012). Interestingly, however, and unlike RSC, the PHC was not sensitive to differences between good and poor navigators. We therefore included PHC as a second ROI in our analysis. As in Auger et al. (2012), ROIs were defined using anatomical masks for RSC (BA 29/30) and PHC that had been delineated by an experienced researcher not involved in the project on an averaged structural MRI brain scan from a different set of *n* = 30 participants, and guided by Duvernoy and Bourgouin (1999), Insausti et al. (1998), and Vann et al. (2009). As a control, we also examined a region not previously implicated in processing specific item features, the motor cortex (Auger et al., 2012).

### 2.6 Data analysis

In the first instance, we sought to ascertain if our ROIs were more engaged by permanent than non-permanent items, now that multiple rather than single items were being viewed. If so, this would accord with results from previous work (Auger et al., 2012). We used the MarsBaR toolbox (http://marsbar.sourceforge.net/) to extract the principal eigenvariate of the fMRI BOLD responses within the anatomically defined ROI masks for each subject. Responses within the RSC and PHC were significantly greater for stimuli containing 4 permanent items than for those containing none (collapsed across hemispheres, BOLD response in arbitrary units, mean difference in RSC .45, SD 1.05; *t*_31_ = 2.42, *p* < .02; mean difference in PHC .55, SD .77; *t*_31_ = 4.02, *p* < .0001). However, using this mass-univariate approach, there were no significant correlations between responses in either of the regions and the number of permanent items in view (RSC: mean *r* = .13, SD .47; not significantly different from 0: *t*_31_ = 1.577, *p* = .1; PHC mean *r* = .17, SD .51; not significantly different from 0: *t*_31_ = 1.937, *p* = .06).

We then progressed with another method, MVPA, that has been found to be more sensitive in some circumstances to stimulus representations (Chadwick et al., 2012; Haynes & Rees, 2006; Norman et al., 2006). We used this to assess whether patterns of activity in RSC and PHC contained sufficient information to decode the number of permanent items present for any given trial (for all 32 participants), with five possible options: 0, 1, 2, 3 or 4 permanent (i.e., never moving) items in view. As in previous studies (Bonnici et al., 2012; Chadwick, Hassabis, & Maguire, 2011, Chadwick et al., 2012), we first performed feature selection, the purpose of which is to reduce the set of features (in this case, voxels) in a dataset to those most likely to carry relevant information. This is effectively the same as removing voxels most likely to carry noise, and is a way of increasing the signal-to-noise ratio (Guyon & Elisseeff, 2003). Having identified participant-specific voxels within the ROIs which provided the greatest amount of permanence information, the final classification used only these most informative voxels. For the overall classification procedure, data from 2 sessions were used for feature selection, with the remaining independent third session's data being used only for the final classification in order to avoid so-called “double dipping” (Kriegeskorte, Simmons, Bellgowan, & Baker, 2009). The same process was repeated changing which sessions were used for feature selection and the final classification each time; these results were then averaged to provide an overall three-fold cross-validation.

During both the feature selection and final classification we used a standard cross-validation technique (Duda, Hart, & Stork, 2001; Hsu & Lin, 2002). Data from a single trial was assigned as the *test* trial, with all remaining trials allocated as *training* trials. A linear support vector machine (SVM) using the LIBSVM implementation (Chang & Lin, 2011) with fixed regularization hyperparameter *C* = 1, was first trained using the *training* data and subsequently tested upon the *test* trial. This process was repeated in turn so that each trial was used as the designated *test* trial once. Classification accuracy was taken as the proportion of correct ‘guesses’ made by the SVM across all the trials.

We used a multivariate searchlight strategy for the feature selection (Kriegeskorte, Goebel, & Bandettini, 2006), which determines the information present in the local space surrounding each voxel. For each voxel within the given ROIs, a small ‘local environment’ was defined as a surrounding sphere of radius 3 voxels which remained within the ROI. This radius was chosen because previous demonstrations of decoding using the searchlight method used radius three (Bonnici et al., 2012; Chadwick, Hassabis, Weiskopf, & Maguire, 2010; Hassabis et al., 2009; Kriegeskorte et al., 2006). Each of the voxel ‘local environments’ were then assessed for how much permanence information they contained using a linear SVM with the procedure described above. This produced a percentage accuracy value for each voxel within an ROI. The voxels with the maximal accuracy value were selected to be used in the final classification.

Overall, this procedure produced an accuracy value for each ROI based on the percentage of trials that were correctly classified. The set of accuracy values across the group of participants was then tested against chance level of 20% (as there were five possible options) using a one-tailed *t*-test. Other comparisons (e.g., between item features) were made using ANOVAs, the results of which were further interrogated using two-tailed *t*-tests. All statistical tests were performed using SPSS version 20. In order to test the specificity of any permanence representation in these regions, we conducted new analyses using the exact same procedure (including new rounds of feature selection) to analyse the size and visual salience of items depicted in stimuli.

### 2.7 Good versus poor navigators

We then divided participants into 16 good and 16 poor navigators by taking a median split of participants' scores on the SBSOD questionnaire administered in the post-scan debriefing session. When comparing good and poor navigators, feature selection was not appropriate because this results in different voxels for each participant being used for the final classification, which could be biased by participants' navigation ability. Therefore, in order to compare good and poor navigators in an unbiased fashion, it was necessary to define a set of voxels to be used for classification in all participants. We identified this set of voxels based upon data from a completely independent cohort of participants in our previous fMRI study (Auger et al., 2012); specifically, the voxels which showed increased activity for items with greater permanence (see Fig. 2B in Auger et al., 2012) which fell within the anatomical ROIs for RSC and PHC.

Given that removing feature selection reduces overall classifier accuracy (Guyon & Elisseeff, 2003), we used a 2-way classification in this decoding analysis, asking whether a majority (3 or 4) or minority (0 or 1) of the items in view were permanent. The classifier accuracies across sessions were averaged to give a classification performance value for each participant's ROIs. When interrogating the data, one-tailed *t*-tests were used to compare good and poor navigators, given the previous finding of difference between these groups for item permanence (Auger et al., 2012). Two-way classifications were also performed for the size and visual salience of items, and comparisons made between the good and poor navigators. These analyses (including two-tailed *t*-tests) were carried out on voxels contained within the RSC and PHC anatomical masks which showed increased activity related to size and visual salience of items in Auger et al. (2012) (see their Fig. 2A). In order to test the specificity of any differences identified between the good and poor navigator groups, we also performed identical comparisons when the participants were divided into males and females.

## 3 Results

### 3.1 Behavioural data

During scanning, participants, who were naïve to our interest in item features, engaged in a vigilance task. They performed with a high level of accuracy (mean 88.4%; SD 15.7), showing they focussed on this dot-detection task and maintained attention during the experiment. Performance was similar across each permanence category. Similarly, there was no difference between good and poor navigators on this measure (mean good 88.19%, SD 13.6; poor 88.54%, SD 18; *t*_30_ = −.62, *p* = .95). Vigilance catch trials were removed from the fMRI analysis.

Ratings provided in the post-scan debriefing indicated that participants found the task overall to be easy (1-very easy to 5-very hard: mean 1.8, SD .7). They also found it easy to view the four items in each stimulus separately without linking them together into a scene (1-very easy to 5-very hard: mean 1.8, SD .9).

For some analyses, the 32 participants were split into good and poor navigator groups (*n* = 16 in each) by taking a median split of SBSOD (Hegarty et al., 2002) scores that were provided in the post-scan debriefing (good group mean 5.6, SD .48; poor group mean 3.9, SD .90; maximum score = 7). The two groups had similar numbers of males (9 good and 7 poor navigators) and females (7 good and 9 poor navigators) and were also similar in age (mean age good navigators 23.6 years, SD 2.03; poor 23.4 years, SD 2.96; *t*_30_ = .278; *p* = .78), how easy/difficult they found the task overall (mean difficulty rating out of 5: good 1.8, SD .91; poor 1.8, SD .54; *t*_30_ = .000; *p* = 1.0), how easy/difficult they found it not to link the items together into a scene (mean difficulty rating out of 5: good 2.0, SD 1.03; poor 1.7, SD .70; *t*_30_ = 1.000; *p* = .33), their visual memory as measured by the delayed recall of the Rey–Osterrieth Complex Figure (good 23.6, SD 5.84; poor 23.4, SD 4.50; *t*_30_ = .119; *p* = .91; maximum score = 36), and their visual information processing ability and abstract reasoning skills as measured by the Matrix Reasoning sub-test of the Wechsler Abbreviated Scale of Intelligence (mean scaled score good 13.0, SD 2.10; poor 12.5, SD 2.22; *t*_30_ = .655; *p* = .52; maximum score = 19). We also carried out a voxel-based morphometry analysis (VBM; Ashburner & Friston, 2000, 2005) and found no structural brain differences between the groups anywhere in the brain, including PHC and RSC.

### 3.2 Eye-tracking data

Robust eye-tracking data were collected from 30 of the 32 participants. We defined 4 areas of interest within the visual field which corresponded to the locations of the 4 grey boxes within which items appeared on each stimulus. We calculated the proportion of each 6 sec trial which participants spent looking at each of these 4 areas. We found no biases in terms of where the participants looked (mean time per trial spent looking at each location: top left 1.32s, SD .43; top right 1.26s, SD .41; bottom left 1.27s, SD .43; bottom right 1.31s, SD .39, other screen locations .89s, SD .42; *F*_3,27_ = .290, *p* = .83). There were also no significant differences between good and poor navigators in the time spent looking at items in the 4 locations (*F*_3, 26_ = .215, *p* = .89). We also considered whether there were any systematic differences in the type of item participants first looked at after stimuli appeared on screen to see if, for example, permanent items were more commonly viewed first. There were no differences in the proportion of permanent items looked at first, for all subjects (permanent 49.7%, not permanent 50.3%; tested against 50% chance: t_29_ = −.386; *p* = .70) and when comparing good and poor navigators (t_28_ = −.891; *p* = .38).

### 3.3 MVPA

**Fig. 2:**
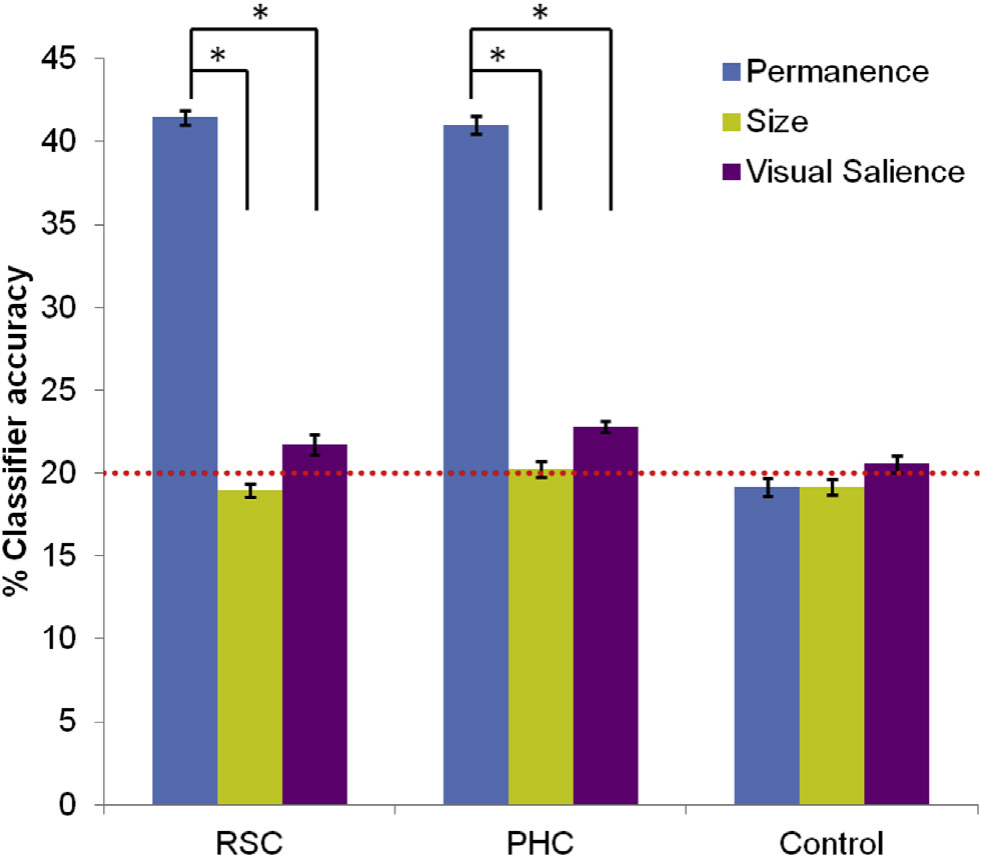
MVPA results. Mean classifier accuracy values for all 32 participants +/− 1 SEM, collapsed across hemispheres. Results for decoding of permanence (blue), size (yellow) and visual salience (purple) are shown for RSC, PHC and a control region (motor cortex). For RSC and PHC, five-way classification of the number of permanent items within each stimulus was not only significantly above chance (which was 20% – red dashed line) but also significantly greater than that for size and visual salience. *p < .05.

**Fig. 3:**
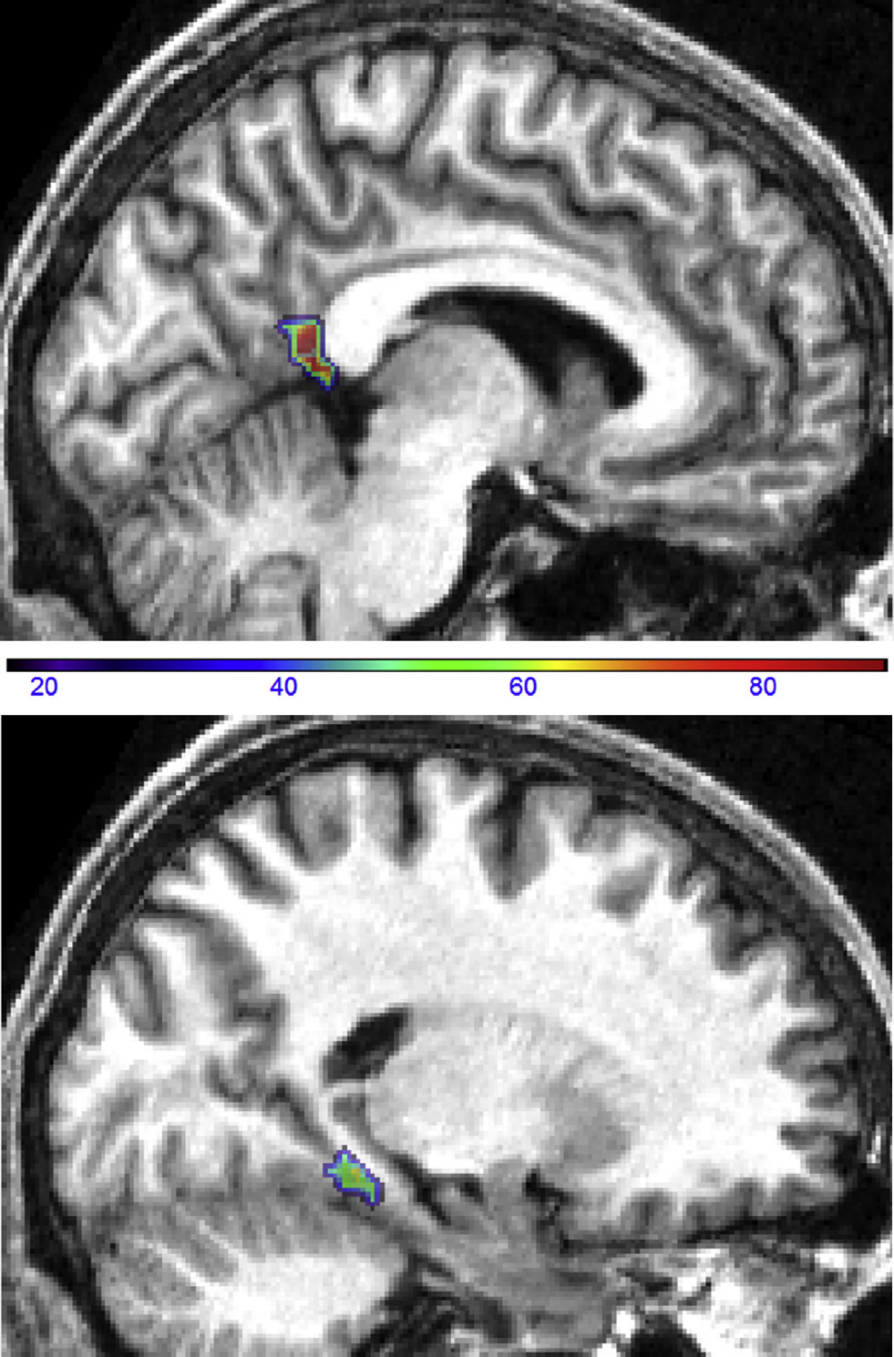
Voxels carrying the greatest amount of permanence information. In these heatmaps, shown on the structural MRI scan of one participant chosen at random, the colours represent the percentage of all 32 subjects in which each voxel was identified by feature selection to carry large amounts of permanence information; RSC top panel, PHC lower panel.

We found no significant differences between classifier accuracies in the two hemispheres (*F*_2,30_ = .990, *p* = .38) and so we report results collapsed across hemispheres. We first examined whether patterns of activity across voxels in RSC could be used to decode the number of permanent items (0–4) in view for a given trial. We found that decoding was possible, significantly above chance (chance = 20%; mean classifier accuracy 41.4%, SD 2.41; *t*_31_ = 50.3, *p* < .0001; Figs. 2 and 3). By contrast, it was not possible to decode the size of the items in view from patterns of activity across voxels in RSC (mean classifier accuracy 19.0%, SD 2.45; *t*_31_ = −2.4, *p* = .02 – note that this is just below chance). Classification of the visual salience of items was significantly above chance (mean classifier accuracy 21.7%, SD 3.42; *t*_31_ = 2.89, *p* = .007; Fig. 2). Notably, however, and as is apparent from Fig. 2, classification accuracy within RSC was significantly greatest for permanence than for the other landmark features (*F*_2, 30_ = 608, *p* < .0001; permanence versus size *t*_31_ = 34.5, *p* < .0001; permanence versus visual salience *t*_31_ = 26.0, *p* < .0001).

We next considered our second ROI, the PHC, which in the previous study of landmark features showed increasing engagement the more permanent the landmarks (Auger et al., 2012). Decoding of permanence category was possible from activity across voxels in the PHC (mean classifier accuracy 41.0%, SD 3.07; *t*_31_ = 38.7, *p* < .0001; Figs. 2 and 3). As with RSC, it was not possible to decode size (mean classifier accuracy 20.2%, SD 2.59; *t*_31_ = .5, *p* = .6), while classification of the visual salience of items was significantly above chance (mean classifier accuracy 22.8%, SD 1.98; *t*_31_ = 8, *p* = .001; Fig. 2). As before (see Fig. 2), classification accuracy within PHC was significantly greatest for permanence than for the other landmark features (*F*_2, 30_ = 500, *p* < .0001; permanence versus size *t*_31_ = 30.3, *p* < .0001; permanence versus visual salience *t*_31_ = 27.8, *p* < .0001). Direct comparison of RSC and PHC showed no significant region by feature type interaction across all subjects (*F*_2, 30_ = 1.89, *p* = .17) [or in good (*F*_2,__14_ = .66, *p* = .53) or poor (*F*_2,__14_ = .74, *p* = .49) navigators separately]. To summarise, we found that RSC and PHC tracked the amount of permanent items in view, but not item size or visual salience.

We also examined classifier accuracy values in control (i.e., not thought to be item feature-related) cortical regions in the left and right motor cortex. Classification accuracy was not above chance for permanence (collapsed across left and right hemisphere, mean classifier accuracy = 19.2%, SD = 3.2; *t*_31_ = −1.48, *p* = .15), size (mean classifier accuracy = 19.1%, SD = 2.7; *t*_31_ = −1.86, *p* = .07) or visual salience (mean classifier accuracy = 20.5%, SD = 2.8; *t*_31_ = 1.12, *p* = .27). This shows that our classification analysis was not biased towards invariably producing above chance accuracies for permanence.

### 3.4 Good versus poor navigators

**Fig. 4:**
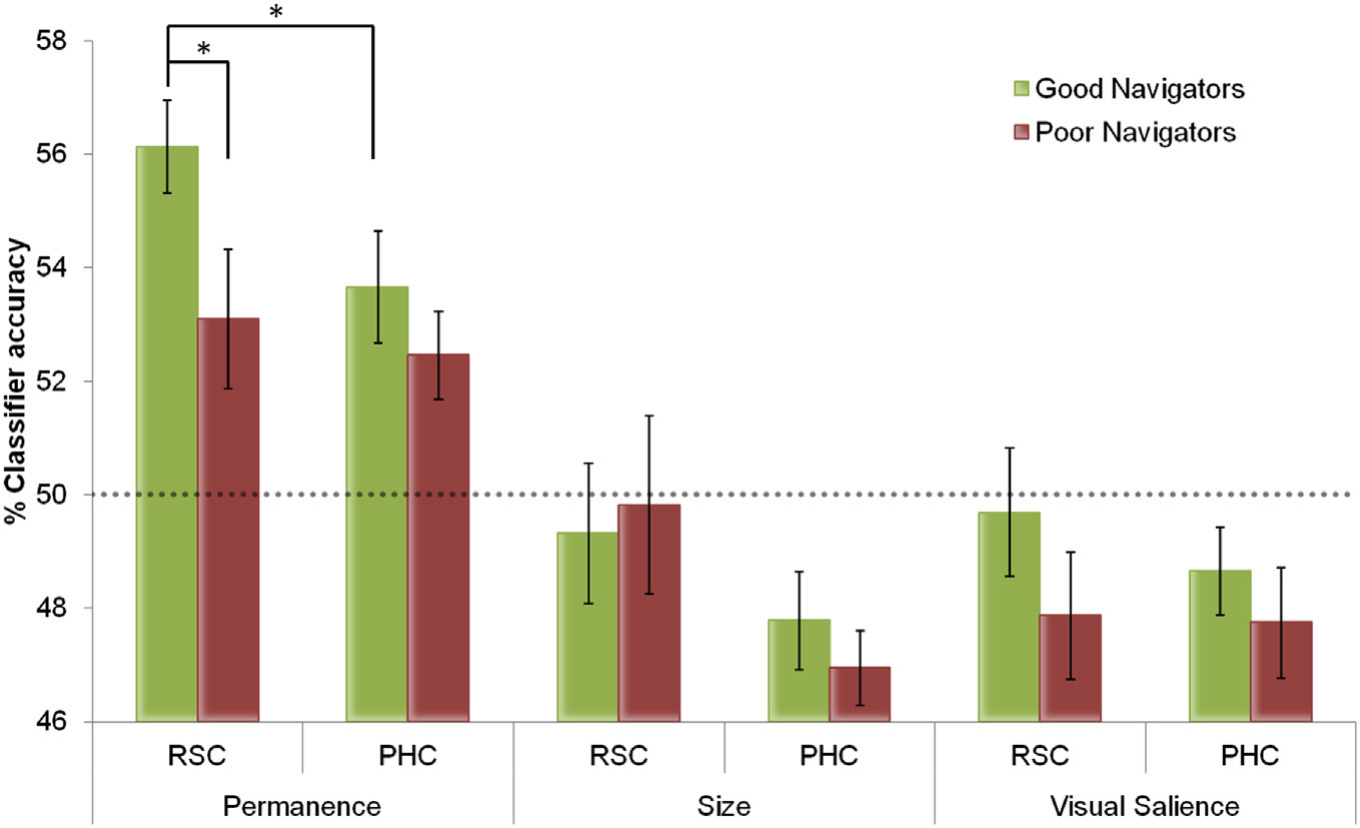
Results for good and poor navigators. Mean MVPA results +/− 1 SEM in good (green) and poor (red) navigators for each of the 3 item features in RSC and PHC. Permanence was the only feature that could be decoded significantly above chance (which was 50% – grey dashed line). Additionally, classification within the RSC of good navigators was significantly greater than that of poor navigators. RSC also contained significantly more permanence information than PHC within good navigators. *p < .05.

As in the previous analysis we found no significant differences between classifier accuracies in the two hemispheres (*F*_2,30_ = .384, *p* = .68) and so we report results collapsed across hemispheres. We directly compared classifier accuracies between good and poor navigators to look for any differences in the amount of permanence information encoded in their neural responses in RSC. Significantly better classification of permanence was possible in the RSC of good (good mean 56.1% SD 3.3) compared to poor navigators (poor mean 53.1% SD 4.9; *t*_30_ = 2.056, *p* < .024; Fig. 4). By contrast, there were no differences in classifier accuracies between good (good mean 53.7% SD 4.0) and poor navigators for PHC (poor mean 52.5% SD 3.1; *t*_30_ = .956, *p* = .17). This indicates that in RSC but not PHC there was significantly more permanence information in the patterns of neural responses of good navigators compared to poor navigators. Other analyses also showed that within good navigators there was significantly better decoding of permanence in RSC compared with PHC (*t*_15_ = 1.82, *p* = .04), while for poor navigators there was no such regional difference (*t*_15_ = .045, *p* = .33; Fig. 4). We performed similar comparisons between good and poor navigators for size and visual salience. Mean classifier values: for size – RSC: good mean 49.3% SD 4.9; poor mean 49.8% SD 6.3; PHC: good mean 47.8% SD 3.4; poor mean 47.0% SD 2.6, and for visual salience – RSC: good mean 49.7% SD 4.5; poor mean 47.9% SD 4.5; PHC: good mean 48.7% SD 3.1; poor mean 47.7% SD 3.9. There were no differences between the two groups for either feature in RSC or PHC (all *t* ≤ 1.14, *p* > .26) or within each group (all *t* ≤ 1.92; *p* > .08). In a set of control analyses, we also compared males and females for permanence, size and visual salience, in both RSC and PHC, but found no significant differences based upon sex.

To summarise, there were no demographic, cognitive or structural brain differences between the good and poor navigators. Neither were there any differences in decodable information in RSC and PHC about the size or visual salience of items in view. Furthermore, there was no difference in the ability to predict whether a majority or minority of viewed items were permanent based upon patterns of activity across voxels in PHC. The only difference between the two groups concerned the accuracy with which it was possible to predict whether stimuli containing a majority or minority of permanent items were in view, with good navigators having significantly more information about the number of permanent items in view in their RSC.

## 4 Discussion

In a previous fMRI study, we found that the RSC responded in a highly selective manner to only the most permanent items when stimuli were presented singly (Auger et al., 2012). Here we found that in a situation that was more akin to real life, with multiple items in view, the RSC coded for the specific number of permanent items contained in a visual array. Moreover, this effect was selective, and was not apparent for other item features such as size and visual salience. This detailed tracking of the amount of permanent items in view was echoed in the PHC, although the two brain structures diverged when participants were divided into good and poor navigators. There was no difference in the responsivity of the PHC between the two groups, while significantly better decoding of the number of permanent items in view was possible from patterns of activity in the RSC of good compared to poor navigators. Within good navigators, the RSC also facilitated significantly better prediction of landmark permanence than the PHC. Overall, these findings suggest that the RSC in particular could be concerned with precisely coding permanent stable items in the environment, and opens up the possibility that this might be a prerequisite for effective navigation.

### 4.1 RSC representation of permanent items

Following our previous findings reported in Auger et al. (2012), the exact parameters within which the RSC operates when responding to item permanence were unclear. Specifically, we wondered whether the RSC response merely reflects the binary presence or absence of something permanent, or whether it contains information about every individual permanent item. The current results show that the RSC does not merely execute a general response to item permanence. Instead, it has a more nuanced representation of the exact number of permanent items that are in view, a fact which only became apparent when using the more sensitive method of MVPA. This throws new light on the mechanism at play within the RSC, and reveals a means by which the RSC could play a crucial role in laying the foundations of our allocentric spatial representations of the environment, which are dependent in the first instance on multiple stable landmarks (Siegel & White, 1975). It is also interesting to note that this response to item permanence was automatic. The participants were naïve to our interest in item features and instead performed an incidental vigilance task that involved searching the images for a blue dot which would occasionally appear on an item. Given the importance of being able to code for stable items in an environment, it is perhaps not surprising that such processing is implicit and automatic, as has been shown for the detection of other components such as animals or vehicles within scenes in the absence of direct attention (Fei Fei, VanRullen, Koch, & Perona, 2002).

One might argue that our results could have been influenced by factors other than permanence, for example, item size (Konkle & Oliva, 2012); after all, big items tend to move less and be more stable. However, not only did we ensure that a range of real-world size values were represented within each permanence category, but the stimuli were designed such that real-world size could be analysed across five categories in a similar manner to permanence. Yet classifiers operating on voxels in the RSC were unable to predict item size. In a similar vein, the decoding of visual salience of the items from activity in RSC was significantly worse than for permanence. Our eye-tracking data confirmed that there were no biases in terms of where and for how long subjects looked within the visual arrays, and this included their viewing of permanent items. Contextual effects (Bar, 2004; but see Mullally & Maguire, 2011) are also an unlikely explanation of our findings because stimuli were presented without any explicit contexts – each item within a stimulus was displayed on a white background inside a grey outline (Fig. 1). Even if subjects had somehow implicitly processed the typical context for each item, the disparate nature of the four items in an array would likely have given rise to conflicting contextual information, thus adversely affecting classifier performance. The permanent items were all perceptually and semantically different, not just in terms of their size and visual salience, but also more generally; they included disparate items such as buildings, trees, telephone boxes, small fixed garden ornaments. Given that the only unifying property between the permanent items was this high level feature, it is perhaps surprising that the magnitude of classifier accuracy was so great, being very significantly above the level of chance. This reinforces the functional importance of the representation of permanence, and underscores the selective response of the RSC to this item feature.

Subjects were also instructed not to link the items that comprised an array together into a scene, and confirmed in post-scan ratings they had not done so, rather they had viewed them as separate entities. This, along with the finding of the RSC responding specifically to the number of permanent items, does not fit easily with the idea that RSC (and PHC) processes the three dimensional geometric structure of scenes (Epstein, 2008; Epstein & Ward, 2010; Henderson, Larson, & Zhu, 2008; Henderson, Zhu, & Larson, 2011) or that RSC contains no information about objects (Harel, Kravitz, & Baker, 2012). Our results are more consistent with a proposal from MacEvoy and Epstein (2011) that a unified representation of whole scenes arises from parallel processing of individual objects within them. Here, we provide further evidence for the simultaneous processing of multiple items, but extend this by identifying a mechanism whereby the properties of local items within a space are key (Mullally and Maguire, 2011), with their permanence seeming to be particularly important. The increased activity in RSC in response to scenes with an explicit three dimensional structure that have been reported frequently in the literature could reflect the presence of multiple permanent items within them. This accords with our previous proposal (Auger et al., 2012) that the RSC's contribution may be to provide input regarding permanent items upon which other brain areas (e.g., the hippocampus) can then build effective spatial and scene representations that are central to episodic memories, imagining the future and spatial navigation (Hassabis & Maguire, 2007; Maguire & Mullally, 2013; Ranganath & Ritchey, 2012; Schacter et al., 2012). The specific nature of RSC input was unclear. Our demonstration here that RSC represents every individual permanent item that is in view, shows that the information it represents and makes available is detailed and precise.

### 4.2 Good versus poor navigators

It is particularly interesting that the information available in the multi-voxel activity patterns in RSC related significantly to the efficacy of participants' spatial navigation. We previously found poor navigators to be less reliable at characterising permanent, ‘never moving’, items compared to good navigators, and also to have reduced responses in RSC when viewing permanent items in isolation (Auger et al., 2012). The present study extends these finding by showing that despite the two groups being closely matched on a range of demographic, cognitive and structural brain measures, poor navigators had less informative neural responses about the permanence of multiple items that were in view simultaneously. Furthermore, the difference in engagement between good and poor navigators was specific to RSC, and not apparent in PHC; while within good navigators, the RSC facilitated significantly better prediction of landmark permanence than the PHC. It seems, therefore, that while RSC and PHC play a role in processing permanent items, only responses in RSC seem to relate to behavioural performance. This may also help to explain the spatial disorientation that is typically associated with bilateral lesions to the RSC (Maguire, 2001b; Vann et al., 2009) and in Alzheimer's disease where RSC hypometabolism is observed at the earliest stages (Minoshima et al., 1997; Nestor et al., 2003; Pengas et al., 2010; Villain et al., 2008). An inability to orientate oneself in space might arise from unreliable landmark permanence representations in RSC, analogous to that observed here in the poor navigator group.

### 4.3 Future directions

While we have drilled down into RSC function here and uncovered a potential concrete explanation for its engagement in a range of cognitive functions that involve spatial contexts and scenes, clearly much remains to be understood. Future work will need to examine this RSC-permanence hypothesis in relation to real-world scenes. The cellular mechanisms within RSC that support the coding of item permanence in complex visual arrays or scenes also need to be investigated. Studies in humans (Foster, Dastjerdi, & Parvizi, 2012) and non-humans (Yoder, Clark, & Taube, 2011) have yet to explicitly examine the direct effects of permanence on neural responses. We speculate that the mechanism for registering permanent items may involve head direction cells, which are present in the RSC (Chen, Lin, Green, Barnes, & Mcnaughton, 1994; Cho & Sharp, 2001), perhaps anchoring themselves to each permanent item. It will also be interesting for future studies to explore how the RSC comes to learn about item permanence in the first place, and to investigate whether permanence more generally, i.e., that is not necessarily tied to absolute spatial locations, is also coded by the RSC.
